# Supplementary material for: The causal relationship between the human gut microbiota and pyogenic arthritis: a Mendelian randomization study
Source: Front Cell Infect Microbiol. 2024 Nov 26;14:1452480. doi: 10.3389/fcimb.2024.1452480 (PMC11629706; doi:10.3389/fcimb.2024.1452480)
Supplement: Supplementary file 1 [file DataSheet1.zip › mendelian test/ebi-a-GCST90027479.csv_forest.pdf]

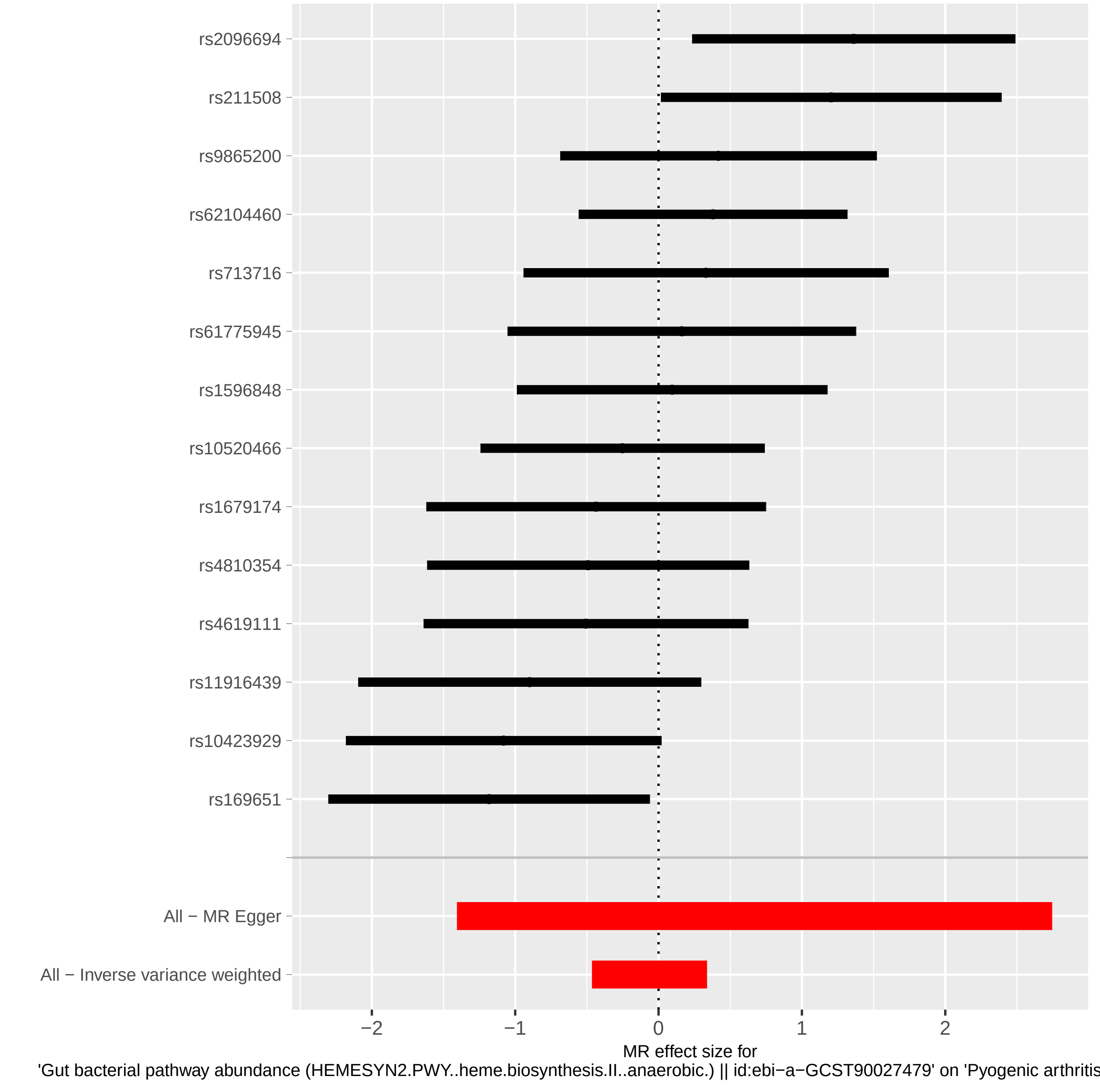
rs2096694
rs211508
rs9865200
rs62104460
rs713716
rs61775945
rs1596848
rs10520466
rs1679174
rs4810354
rs4619111
rs11916439
rs10423929
rs169651
All − MR Egger
All − Inverse variance weighted
−2
−1
0
1
2
MR effect size for
'Gut bacterial pathway abundance (HEMESYN2.PWY..heme.biosynthesis.II..anaerobic.) || id:ebi−a−GCST90027479' on 'Pyogenic arthritis
